# Convolutional Neural Networks with 3D Input for P300 Identification in Auditory Brain-Computer Interfaces

**DOI:** 10.1155/2017/8163949

**Published:** 2017-11-07

**Authors:** Eduardo Carabez, Miho Sugi, Isao Nambu, Yasuhiro Wada

**Affiliations:** Department of Electrical Engineering, Nagaoka University of Technology, 1603-1 Kamitomioka, Nagaoka, Niigata 940-2188, Japan

## Abstract

From allowing basic communication to move through an environment, several attempts are being made in the field of brain-computer interfaces (BCI) to assist people that somehow find it difficult or impossible to perform certain activities. Focusing on these people as potential users of BCI, we obtained electroencephalogram (EEG) readings from nine healthy subjects who were presented with auditory stimuli via earphones from six different virtual directions. We presented the stimuli following the oddball paradigm to elicit P300 waves within the subject's brain activity for later identification and classification using convolutional neural networks (CNN). The CNN models are given a novel single trial three-dimensional (3D) representation of the EEG data as an input, maintaining temporal and spatial information as close to the experimental setup as possible, a relevant characteristic as eliciting P300 has been shown to cause stronger activity in certain brain regions. Here, we present the results of CNN models using the proposed 3D input for three different stimuli presentation time intervals (500, 400, and 300 ms) and compare them to previous studies and other common classifiers. Our results show >80% accuracy for all the CNN models using the proposed 3D input in single trial P300 classification.

## 1. Introduction

Brain-computer interfaces (BCI) offer a way for people to communicate with devices using their brain. Although the applications and environments in which BCI have been explored are numerous, here we focus on their potential supporting role for people with muscle movement limitations.

Some BCI use event-related potentials (ERP) to link a person's brain to the actuator or device the person intends to interact with. ERP are brain activity patterns that can be measured by electroencephalography (EEG). Among the many ERP, we used P300 for this study. P300 is the positive deflection expected between 250 and 700 ms after the BCI user identifies an irregular (expected) cue among regular ones in an experimental setup. This way of presenting stimuli to the BCI user is known as the oddball paradigm. P300 can be elicited through the oddball paradigm using different stimuli (e.g., sound or image). BCI applications and experiments involving EEG, P300, and image stimuli that focus on people with motor disadvantages have been widely explored and successfully developed in the past [1–3].

For this study, we used sound stimuli to elicit P300 through the oddball paradigm. Although images have been successfully used for such tasks, their use requires that the subjects (who might have physical disabilities) retain control of their eyes and some face and head muscles as well. However, that is not the case for blind people who have lost their ability to see or were never sighted, or for patients with complete locked-in syndrome, who are not in control of their eye movements. By using sound stimuli, we believe that a more portable BCI can be developed, which is suitable for those who cannot receive visual stimuli or simply prefer to dedicate their vision to other tasks.

Once P300 is elicited, the BCI should be able to recognize it and classify it as such. For this purpose, we used several convolutional neural network (CNN) structures. CNN represent a specific topology of a multilayer perceptron (part of the artificial neural network (ANN) family). Like many other machine learning models, CNN have been used for classification purposes with satisfactory results in different applications [4–7].

Unlike many types of ANN, CNN can handle two- or three-dimensional (2D or 3D) inputs without mapping data onto a one-dimensional (1D) vector, which can be a cause of information loss depending on the nature of the data. Data mapping is common in BCI applications, but as studies show that eliciting P300 causes stronger brain activity in certain brain regions, maintaining both spatial and temporal EEG information when making the CNN input might be key to achieving higher accuracy in P300 classification. With this in mind, we propose a novel 3D input for the CNN. Our approach avoids the information loss that comes with data mapping and allows main CNN operations (convolution and pooling) to take place without the limitations described in other studies [8].

We use our proposed 3D input to test 30 different CNN structures for P300 classification. The CNN structures varied from each other by the kernels (patches) used during the convolution or pooling processes. We also used different pool strides to cause or avoid overlapping, depending on the case, as this has been reported to improve the CNN performance in some applications [9].

The following sections of this work are organized as follows: in Section 2, we explain in detail the experimental setup used to produce and process the dataset used. Further, the general CNN structure and details regarding the shape of the proposed 3D input are presented in Section 3. Finally, in Sections 4 and 5, we discuss our results, comparing them to those obtained in other similar studies and also presenting the performance of other common classifiers used in this context.

## 2. Dataset

### 2.1. Experimental Setup

**Figure 1:**
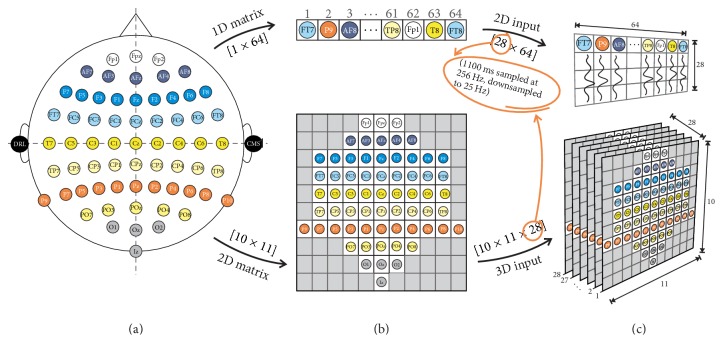
The different steps of input construction: (a) The experimental EEG channel layout. (b) EEG channel matrix disposition to form 2D and 3D inputs (upper and lower images, resp.). Gray cells contain no information. (c) Usual 2D input shape and proposed 3D input shape following our considerations (upper and lower images, resp.).

The dataset used for this study corresponds to evoked P300 waves from nine healthy subjects (8 men, 1 women) obtained using an auditory BCI paradigm. A digital electroencephalogram system (Active Two, BioSemi, Amsterdam, Netherlands) was used to record brain activity at 256 Hz. The device consists of 64 electrodes distributed over the head of the subjects by a cap, using the configuration shown in Figure 1(a). This study was approved by the ethics boards of the Nagaoka University of Technology. All subjects signed consent forms that contained detailed information about the experiment and all methods complied with the Declaration of Helsinki.

**Figure 2:**
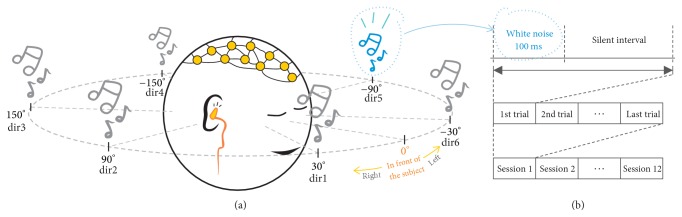
(a) Position representation for the six virtual directions with respect to the subject. (b) Conformation of the 12 sessions all 9 subjects took part of.

The subjects were presented with auditory stimuli (100 ms of white noise), similar to that performed in [10], using the out-of-head sound localization method presented in [11], so that subjects could hear the stimuli coming from one of six virtual directions via earphones (see Figure 2(a)). Stimuli were followed by a silent interval of time. One stimulus and one corresponding silent interval were referred to as a trial. Three different trial lengths (500, 400, and 300 ms) were used to analyze the impact of the speed of stimuli presentation on the identification of the P300 wave. Each subject completed 12 experimental sessions, each consisting of around 180 trials for a given trial length. On each session, the subjects were asked to focus on only the sound perceived to be coming from one of the six virtual directions, which was called the target direction. The subjects counted in silence with their eyes closed every time they perceived sound being produced from the target direction and ignored the rest. Ideally, this should elicit P300. The target direction rotated from directions 1 to 6, one by one, for sessions 1 to 6 and then repeated in the same order for sessions 7 to 12. The direction in which stimuli were presented was pseudorandomized; therefore for every six trials, sound from each direction was produced at least once and stimuli coming from the target direction were never produced sequentially to avoid overlapping of P300.

### 2.2. Preprocessing and Data Accommodation

Before sorting into training and test sets, EEG data were baseline corrected using a Savitzky-Golay filter from −100 ms before stimulus onset until the end of the trial (i.e., end of the silent period after stimulus offset).

A filtering process was also conducted along all EEG channels using Butterworth coefficients for a bandpass filter with cutoff frequencies of 0.1 and 8 Hz. Next data were downsampled to 25 Hz (approximately a tenth of the original size). Data were downsampled as the original size would result in longer processing and training/testing times. Similar downsampling can be found in [10]. Nonaveraged trials were used for this study.

As each subject performed 12 experimental sessions (see Figure 2(b)), with around 180 trials in each of them, data collection for each subject consists of approximately 2160 trials for a given trial length for each subject. Given the pseudorandomized nature of the stimuli production, for each six produced stimuli, one was from the target direction. That stimuli were labeled as the target trial and the rest as nontarget trials. Consequently, of nearly 2160 trials, each subject was expected to produce around 360 target trials as a result of 12 sessions (i.e., a sixth of them), while the remaining are nontarget trials. In this case, the target direction is not particularly relevant, as independently of where the target direction is located, perceiving stimuli correctly from that direction should elicit P300. What is important is to determine is whether the user can differentiate among the six virtual directions and that focusing on one of them and perceiving sound from it are possible with the proposed experimental setup.

Training and test sets were generated for each subject on a given trial length using only that subject's data. To generate the training and test sets for each subject, first we shuffled the target trials with the same happening to the nontarget trials. Next, we distributed half of the target trials in each set with the same applying for nontarget trials. This resulted in training and test sets for each subject containing around 1100 trials each, with approximately 180 target trials and 900 nontarget trials in each set.

**Figure 3:**
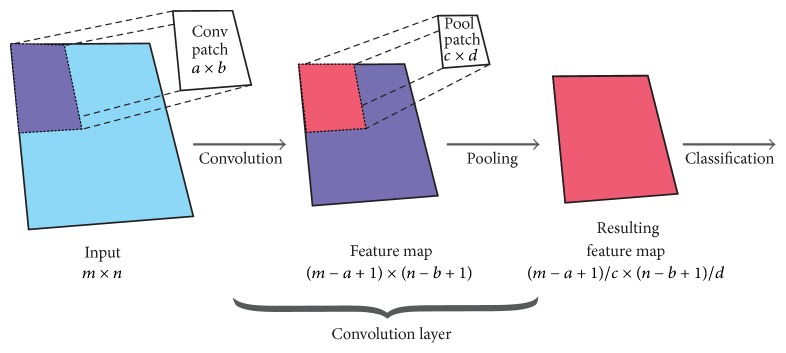
General structure of a CNN. Overlapping is not shown in this figure. Default pooling stride is being applied.

As can be seen in Figure 3(c), regardless of the trial length, the proposed input consisted of 1100 ms of recorded brain activity after stimulus onset. We consider the same amount of information to fairly evaluate all trial lengths and compare our results to previous work in Section 4.

## 3. Input Shape and CNN Model

### 3.1. 3D Input

For the detection of P300 using EEG, the electrode position is relevant as there are areas where the potential is experienced more strongly [10]. This, however, has not been addressed in previous research, instead mapping the 3D data (position of electrodes and time) into a 2D vector that contains all EEG channel activity during the experiment. This not only causes information loss, but also prevents classifiers such as CNN to be used without special consideration (as observed in [8]).

To avoid information loss and limitations of CNN operations, positions of the 64 electrodes were mapped onto a 10 × 11 matrix (see Figure 1(b)), maintaining their position as close as possible to their real arrangement in the experimental setup. Time information is presented through an extra axis, so the 3D input has the shape shown in Figure 1(c). Cells that do not correspond to an electrode (gray ones) are set to zero in all instances.

In Section 4, we presented a 2D input for performance comparison purposes. In that case, the input has the shape depicted in Figure 1 (upper flow). The preprocessing, data accommodation (train and test set size), and any other considerations made for the 3D input in Section 2.2 also apply for the 2D one.

### 3.2. CNN Model

This particular neural network architecture is a type of multilayer perceptron with feature-generation and a dimension-reduction oriented layer, which together compose a convolutional layer. Unlike other layered-based neural networks, CNN can receive a multidimensional input in its original form, process it, and successfully classify it without a previous feature extraction step. The general structure of the CNN is presented in Figure 3. For our study, we used a 3D input and produce 28 feature maps (one for each time sample). While CNN with layers lacking the pooling process are also possible, the pooling process offers scale invariance for the resulting feature maps. It also helps preventing overfitting and allows reduction of computational complexity of the model by reducing the size of the resulting feature maps, thereby shortening training/test times.

Here, we proposed 30 different CNN models to investigate the impact that different convolution and pool patches have on model performance. The proposed models varied from each other in terms of convolution or pool patch size. The CNN models were implemented using a GeForce GTX TITAN X GPU by NVIDIA in Python 2.7 using the work developed by [12].

Additionally, fixed pooling strides were used as an alternative to the default value, which had the same size as the pool patch, with the purpose of forcing pool patches to overlap (or not) during the pooling process, as this has been reported to improve the CNN performance [9]. For this purpose, we applied fixed pooling strides with the values [1 × 1], [1 × 2], [1 × 3], [2 × 2], and [2 × 3]. While normally the pooling stride is given as an integer value, in the work of [12], used in this study, the pooling stride must be defined as an array of two values, with the first one corresponding to the step(s) taken along the *x*-axis and the second one of those taken along the *y*-axis. The whole input is spanned using this approach, with only the pooling process affected. For the convolution process, the stride is 1. When a pooling stride different than the default one is used, areas where the pooling patch is applied to the feature map can overlap from one application to another, or contrarily certain areas can be skipped depending on the size of the stride and the pool patch. With our proposed pooling strides, we intended to cause overlapping in the application areas to show whether this impacts the CNN performance (as in [9]). We believe this approach could benefit CNN models as spanning the same area more than once with the max pooling approach could pick up the features corresponding to the P300 production as this wave causes stronger activity in specific brain areas. This should create a resulting feature map containing multiple times this part of the feature map, making classification easier.

For a given trial length and pool stride value, 30 CNN models were trained for each subject. As there are nine subjects, three trial lengths, and six pool stride values, a total of 4860 CNN were trained for this research. However, only results showing the average performance of the nine subjects will be presented. Tested convolution and pool patches are summarized in Table 1, as well as their patch number, which will be used to present results in the next section.

Each patch is referenced by a number, starting from 0. All possible patch combinations were tested with the resulting model using particular convolution and pool patches, with a patch code consisting of two digits being presented. The first digit corresponds to the convolution patch and the second one to the pool patch. Therefore, for patch code 24, we are referring to the CNN model that used the [3 × 2] convolution patch and the [2 × 3] pool patch. Given that the tested CNN are numerous, we present a statistical analysis in Section 4.1 implementing ANOVA between the models and the proposed pool strides.

As for the learning rate of CNN, it was set at 0.008 based on preliminary tests. The optimization method we used is the stochastic gradient descent as it has been demonstrated [13] to be beneficial for training neural networks on datasets with large examples, using the mini batch approach (batch size of 100). Classification at the output layer is performed using the softmax function, which produces a label based on the probability of a given example to belong to one dataset class.

To calculate classification accuracy we have to consider that the proportion of target and nontarget trials in the training and test sets was not even. Thus, we used the expression

(1) $$\begin{array}{c} accuracy=\sqrt{\frac{TP}{P}\times \frac{TN}{N}}, \end{array}$$

where TP stands for true positives and reflects the number of correctly classified target examples, and TN stands for true negatives and reflects the number of correctly classified nontarget examples. *P* and *N* represent the total number of examples of target and nontarget classes, respectively, for this case. This expression heavily penalizes poor individual classification in binary classification tasks.

## 4. P300 Identification: Results and Discussion

The results presented next correspond to the average accuracy obtained for the nine subjects in testing of CNN models. The highest and lowest accuracy rates are highlighted in bold and red fonts, respectively. By analyzing the performance obtained using different pooling strategies in the form of different fixed pooling strides (presented in the first column from left to right), it is often observed that some pooling strategies do not offer relevant differences at first glance.

**Figure 4:**
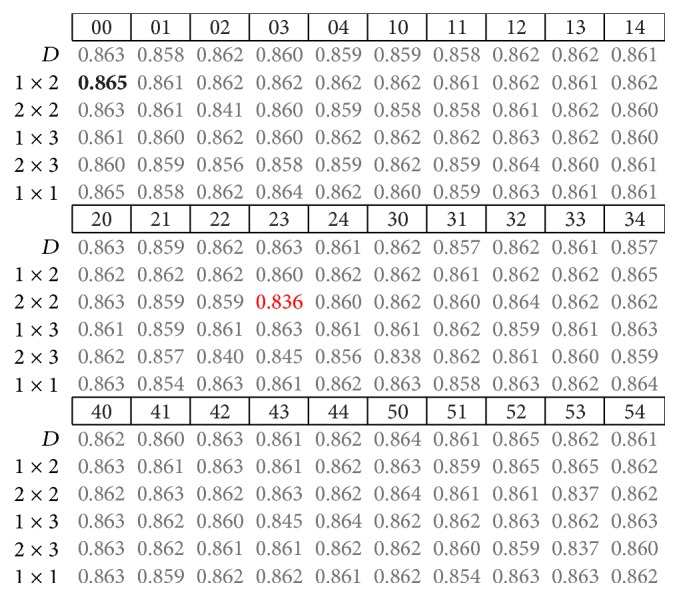
Summary of results from nine subjects in the 500 ms trial interval.

By analyzing the summarized results for the three trial intervals, we found no clear tendency for which model and pool stride offer the highest or lowest accuracies. For instance, in the 500 ms trial interval models (Figure 4), the lowest accuracy was obtained from the model with patch code 23 and [2 × 2] pool stride, while in both the 400 and 300 ms cases, these results were obtained using the model with patch code 30 and [2 × 3] pool stride, which is similar to the 500 ms case.

**Figure 5:**
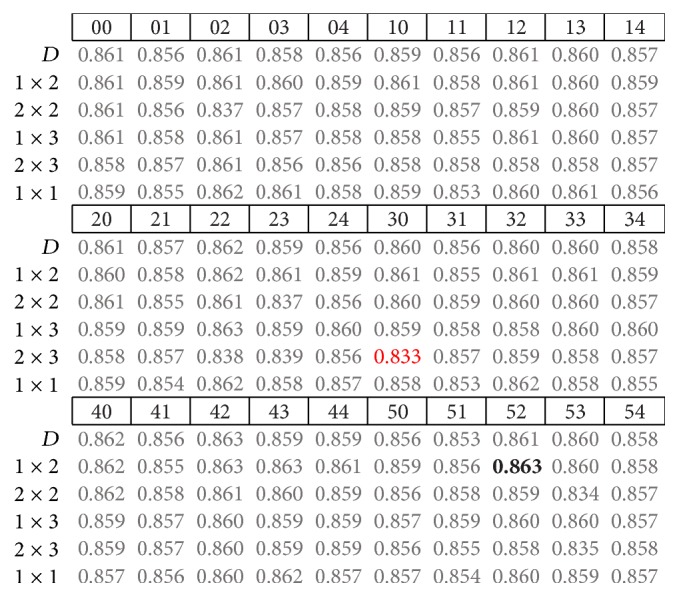
Summary of results from nine subjects in the 400 ms trial interval.

**Figure 6:**
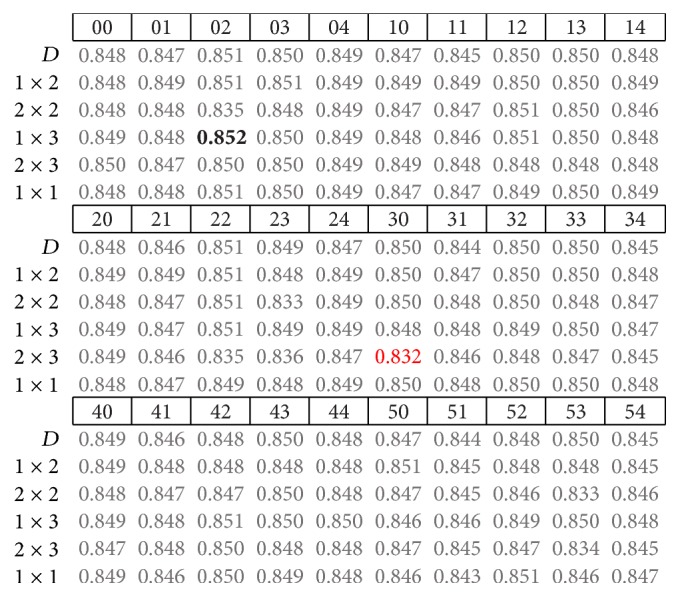
Summary of results from nine subjects in the 300 ms trial interval.

As for the highest accuracy results, there are some similarities in the 400 and 300 ms trial intervals (Figures 5 and 6, resp.). In these cases, the implemented models used pool patch (2) under the [1 × 3] or [1 × 2] pooling strides, which prevent the models from overlapping and are very similar one to each other. For the 500 ms case, pooling stride was also [1 × 2], the same as in the 400 ms trial interval, while the pool patch was different. If we look back at Table 1, we can see that these convolution patches are quite different from each other.

By analyzing Figure 8, it can be noted that even if the results do not vary strongly one from another, there is a clear pattern of improved performance using data from the 500 ms trial length, followed by the 400 and 300 ms ones. This behavior is expected, as faster production of stimuli can cause subjects to fail to identify stimuli coming from the target direction and therefore incorrectly produce the P300 wave. By summarizing the results in this way, we also observed that the [1 × 2] pooling stride offers the best results, at least in the 500 and 400 ms trial length, while the [1 × 3] pool stride is optimal on the 300 ms trial length.

On the other hand, the [2 × 3] pool stride produces the lowest results, without being detrimental. Differences between the highest and lowest pool strides rely on how much overlapping the strides provide. While the [2 × 3] pooling stride prevents some pool patches from overlapping at all or even skipping some areas of the input, the [1 × 3] pooling stride forces most pool patches to overlap. In the study by [13], no differences were reported between performance for approaches with or without overlapping, contrary to the report by [9], where better CNN model performance was achieved using overlapping pool strategies. In our case, we found little to no change between different pooling strategies tested. In the above cited research, it is mentioned that success in applying pooling strategies might depend completely on the nature, shape, and conditions of the used data.

In Table 2, the average values considering all models for each of the three trial lengths are presented next to their corresponding lowest and highest values.

By comparing these numbers, apparently there are no big differences between trial lengths and their highest/lowest values. We believe this lack of variation between the many tested models is the result of the implementation of the 3D input, which, regardless of the speed of the stimuli presentation used in this study, can present the necessary information for correct classification. To support this idea, we tested 4 additional CNN models using the commonly 2D input approach with convolution patches [1 × 4] and [3 × 3]. As for the pool patches, we also tested two, with sizes [1 × 2] and [2 × 2], both with a default pooling stride as our results so far indicate the pooling strategies do not offer significant CNN performance differences. The patches were chosen as they are the same as those used by the CNN models with the best results using the 3D input. Besides the input shape and the convolution and pool patches, the parameters of the CNN models using the 2D input do not differ from the ones presented so far in Section 3.2. The results from the models using the 2D input can be seen in Table 3.

In the case of the results for the models using the 2D input, the difference between models and trial lengths is more easily noticed. While in the results involving the 3D input the difference of the overall highest and lowest accuracy is of about 3%; in the case of the 2D input results, the difference is around 10%. Also, we can see that the convolution and pool patches that consider information from only one channel at a time offer better results than those in which information from multiple channels is considered.

In Table 4 the individual results for each subject considering the models with the highest accuracy for each trial length using both 3D and 2D approaches are shown. The models with highest accuracy are those presented with bold font in Figures 4–6 and Table 3.

The results obtained show individual performance patterns appearing in both approaches in a similar way. For a given trial length, the subject with the highest individual accuracy is the same regardless of the approach (3D or 2D). Although in some cases the accuracy difference between both approaches for a single subject is minimal, all the results from the CNN models using 3D input offered better accuracies.

Besides the difference in the obtained accuracies, the train/test of the 3D input models was also faster than that of the models using the 2D input. The average time of the 3D input models for training/testing a single subject was around 8 minutes, while for the case of the models using the 2D input, around 18 minutes were necessary.

### 4.1. Statistical Analysis

Given that the results obtained so far do not show big differences of the performance of the CNN models whether we consider the models themselves or the trial length in which they were tested, we conducted an analysis of variance (ANOVA) to further examine the results.

First, we checked if applying the different tested models (variation of convolution and pool patch sizes) had a significant impact on the performance of the CNN models. The results are shown in Table 5 for the three trial lengths that were considered for this study.

We found that, for the models using examples from the 500 and 400 ms trial lengths, there were no significant differences, but there were ones for the case of the 300 ms trial length. As the trial length becomes shorter, it becomes harder for users to correctly identify the sound coming from the target direction and in this case the differences between models become clearer.

Next, we present the results for the ANOVA between the tested pool strides in Table 6. In this case, there are significant differences among the implemented pool strides regardless of the trial length. We proposed applying several pooling strides to explore whether by causing or avoiding overlapping during the pooling process the performance of the models improved. In Figure 5 we have the average accuracy for all the models under each pool stride displayed, but the small differences between the results made it difficult to state if they were different enough to make an assessment. Now, the results in Table 6 show that varying the pooling stride to cause overlapping or avoid it significantly impacts the performance of the tested CNN models.

### 4.2. Comparison with Previous Work and Other Classifiers

**Figure 7:**
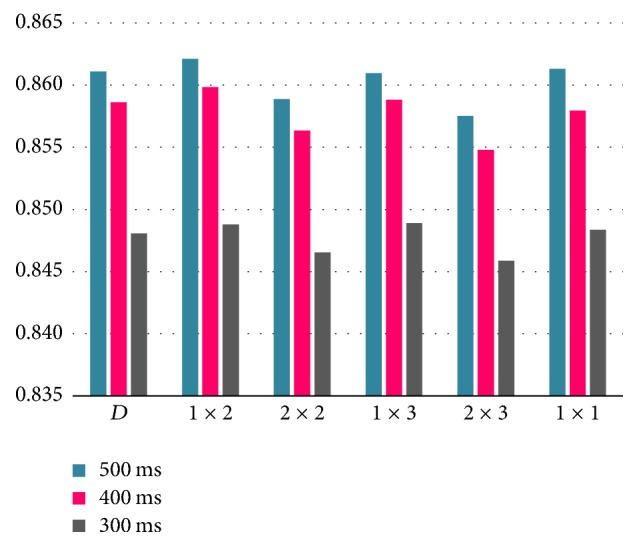
Averaged accuracy rate by pooling stride for three proposed trial intervals.

**Figure 8:**
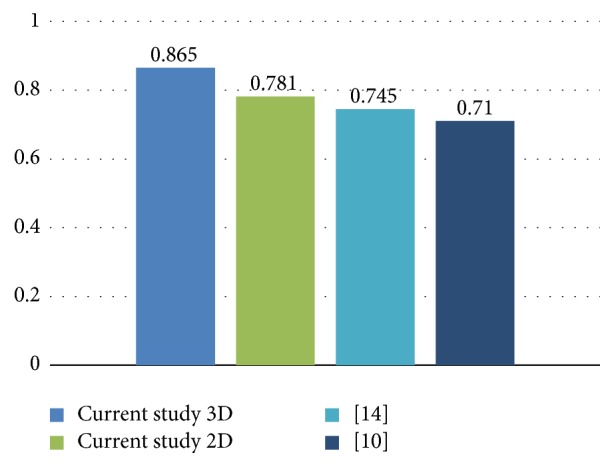
Average accuracy rate for the single trial case. Conditions of experiment and subjects might differ between the studies.

The current results show an improvement of around 15% over the work of [10], from where the experimental setup for this research was borrowed (see Figure 7). The EEG data was obtained also in a similar fashion, enabling the current comparison. Also, this study shares some similarities with that done by [14] which is why we also include it in the comparison. In most cases, it is difficult to make an appropriate comparison due to the differences in the nature of the experiments, subjects, and technologies used and for such reasons, the comparison is only demonstrative. All the results used for comparison in Figure 7 are the ones corresponding to the single trial (also noted as nonaveraged) case. The highest results obtained for the implementation of the 2D input are also included.

We now compare the results from the models using the proposed 3D input with those obtained by using support vector machines (SVM) and Fisher's discriminant analysis (FDA). We chose to use these two classifiers as they are common in this context and the SVM was used in [10].  Table 7 shows the results for comparison of the highest accuracies obtained in the different trial lengths. Details about the SVM and FDA can be found in the appendix.

Both the FDA and the SVM offer accuracies below those from the model using the proposed input.

## 5. Final Comments and Future Work

Through this research we found that it is possible to implement a 3D input shape using EEG data with success for different CNN models that exhibit different pooling strategies based on proposed fixed pooling strides that might cause the models to overlap during the pooling process or avoid it. We hypothesize that using this approach might yield better results compared to the most common approaches which use 2D mapped version of the data. The basis of such thinking lies in the nature of the convolution and pooling processes, which highly depend on the relation between a data point and its surroundings. This lack of variation might point to a better representation of the information given by the proposed 3D input. Also, we found that, for the current study, causing overlapping with fixed pooling stride significantly impacts the performance of the tested CNN models.

The obtained results showed improvement over others seen in similar studies using nonaveraged data. Also, when compared to other classifiers commonly used in this context, the CNN models using the proposed input performed better.

While we believe this was a successful application of a novel input structure, we consider that such construction will perform particularly well when the nature of the data is such that mapping it to simpler representations comes with information loss. For other BCI approaches as well as for other kinds of brain activity readings, this approach might not be the best fit and its application might require a case by case analysis.

With the proposed 3D input we were able to find also a faster way to train/test, as this approach showed taking less time for such tasks than the models using a 2D input. We expect to keep using this input representation to test its limitations and possible new applications in future studies.

## Figures and Tables

**Table 1:** Proposed convolution, pool patches, and pool stride for the current study.

Patch number	Convolution patch	Pool patch	Pool stride
(0)	[3 × 3]	[2 × 2]	Default
(1)	[2 × 2]	[3 × 3]	[1 × 1]
(2)	[3 × 2]	[1 × 2]	[1 × 2]
(3)	[2 × 3]	[1 × 3]	[1 × 3]
(4)	[2 × 4]	[2 × 3]	[2 × 2]
(5)	[1 × 4]		[2 × 3]

**Table 2:** Summary of the highest, lowest, and average accuracies obtained for the CNN models using the 3D input.

	Highest	Lowest	Average
500 ms	0.865	0.836	0.86
400 ms	0.863	0.833	0.858
300 ms	0.852	0.832	0.848

**Table 3:** Proposed convolution and pool patches for the CNN models in which a 2D input was implemented. CP and PP stand for *convolution patch* and *pool patch*, respectively.

CP	PP	Accuracy		
		500 ms	400 ms	300 ms
[1 × 4]	[1 × 2]	**0.781**	**0.768**	**0.734**
	[2 × 2]	0.753	0.716	0.727
[3 × 3]	[1 × 2]	0.766	0.725	0.732
	[2 × 2]	0.724	0.707	0.698

Average		0.756	0.729	0.722

**Table 4:** Results for each subject in those models with the highest accuracy for each trial length considering the CNN models with both the 3D and 2D input approach. The subject's number appears on the first column to the left.

	500 ms		400 ms		300 ms	
	3D	2D	3D	2D	3D	2D
(1)	0.880	0.72	0.877	0.736	0.859	0.685
(2)	0.873	0.823	0.866	0.78	0.878	0.744
(3)	**0.896**	**0.774**	**0.908**	**0.794**	0.859	0.774
(4)	0.828	0.812	0.83	0.766	0.831	0.785
(5)	0.864	0.78	0.851	0.792	0.826	0.697
(6)	0.828	0.808	0.828	0.763	0.825	0.685
(7)	0.881	0.788	0.858	0.752	**0.886**	**0.796**
(8)	0.879	0.792	0.904	0.77	0.847	0.721
(9)	0.863	0.736	0.847	0.76	0.853	0.722

**Table 5:** Results for the ANOVA between the 30 tested models. The critical *F* value is 1.54.

	*F*	*p*	Significant differences
500 ms	1.29	0.159	No
400 ms	1.44	0.08	No
300 ms	1.55	0.047	Yes

**Table 6:** Results for the ANOVA between the 6 implemented pool strides. The critical *F* value is 2.26.

	*F*	*p*	Significant differences
500 ms	4.21	0.001	Yes
400 ms	4.72	0.0004	Yes
300 ms	5.015	0.0002	Yes

**Table 7:** Comparison between the highest accuracies obtained using the proposed 3D input for CNN models, a SVM, and a FDA.

	CNN 3D	SVM	FDA
500 ms	0.865	0.709	0.745
400 ms	0.863	0.711	0.731
300 ms	0.852	0.691	0.707

## Appendix

*SVM*. Analysis was performed using LIBSVM software [15] and implemented in MATLAB (Mathworks, Natick, USA). We used a weighted linear SVM [16] to compensate for imbalance in the target and nontarget examples. Thus, we used a penalty parameter of C+ for the target and C− for the nontarget examples. The penalty parameter for each class was searched in the range of 10^−6^ to 10^−1^ (10^−6^ ≤ 10^*m*^ ≤ 10^−1^;* m*: −6 : 0.5 : −1) within the training. We determined the best parameters as those obtaining the highest accuracy using 10-fold cross-validation for the training. Using the best penalty parameters, we constructed the SVM classifier using all training data and applied it to the test data.

*FDA*. We used a variant of the regularized Fisher discriminant analysis (FDA) as the classification algorithm [14]. In this algorithm, a regularized parameter for FDA is searched for by particle swarm optimization (for details, see [14]) within the training. In this study, we used all EEG channels without selection.
